# Novel homozygous variant in *WISP3* in a family with unrecognized progressive pseudorheumatoid dysplasia

**DOI:** 10.1002/ccr3.2884

**Published:** 2020-05-03

**Authors:** Chandreshkumar Patel, Anas M. Khanshour, David Wilkes, Jonathan J. Rios, Kelly W. Sheff, Lorien Nassi, Carol A. Wise

**Affiliations:** ^1^ Scottish Rite for Children Center for Pediatric Bone Biology and Translational Research Texas Scottish Rite Hospital for Children Dallas TX USA; ^2^ Radiology Department Texas Scottish Rite Hospital for Children Dallas TX USA; ^3^ McDermott Center for Human Growth and Development University of Texas Southwestern Medical Center Dallas TX USA; ^4^ Department of Pediatrics University of Texas Southwestern Medical Center Dallas TX USA; ^5^ Orthopaedic Surgery University of Texas Southwestern Medical Center Dallas TX USA; ^6^ North Texas Genome Center University of Texas at Arlington Arlington TX USA

**Keywords:** juvenile idiopathic arthritis (JIA), progressive pseudorheumatoid dysplasia (PPD), rheumatoid arthritis, whole‐genome sequencing (WGS), *WISP3*

## Abstract

We present the use of whole‐genome sequencing to correctly diagnose progressive pseudorheumatoid dysplasia in patients with atypical clinical and radiologic findings and prior diagnosis of juvenile idiopathic arthritis.

## Graphical abstract

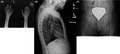

## 1 INTRODUCTION

The clinical hallmarks of inflammatory arthritides are stiffness, effusion, and limited range of motion. Juvenile idiopathic arthritis (JIA), the most common diagnosis of childhood inflammatory arthritis, is an umbrella term that describes a group of heterogeneous disorders that are characterized by arthritis of unknown etiology.^1^ The diagnosis of JIA is challenging due to the heterogeneity and nonspecificity of clinical symptoms and the episodic nature of symptoms. A number of musculoskeletal diseases such as idiopathic multicentric osteolysis, mucopolysaccharidoses, camptodactyly‐arthropathy‐coxa vara‐pericarditis syndrome, and progressive pseudorheumatoid dysplasia can mimic JIA but are noninflammatory in nature.^2^, ^3^ Misdiagnosis can lead to inappropriate and unnecessary treatments.

Technological advances of the past decade have enabled efficient personal genome sequencing,^4^ and next‐generation sequencing of exomes (WES) or genomes (WGS) is emerging as a powerful and effective diagnostic tool in cases with a suspected genetic etiology.^5^ Importantly, WGS can aid diagnosis and lead to more informed clinical decision‐making, as illustrated in a recent study of intensively ill children.^6^ Here, we demonstrate the utility of WGS to inform the differential diagnosis in two siblings that presented with clinical and radiologic symptoms that were consistent with JIA but refractory to standard therapies and negative by panel‐based genetics evaluation.

## 2 PATIENTS AND METHODS

**Figure 1:**
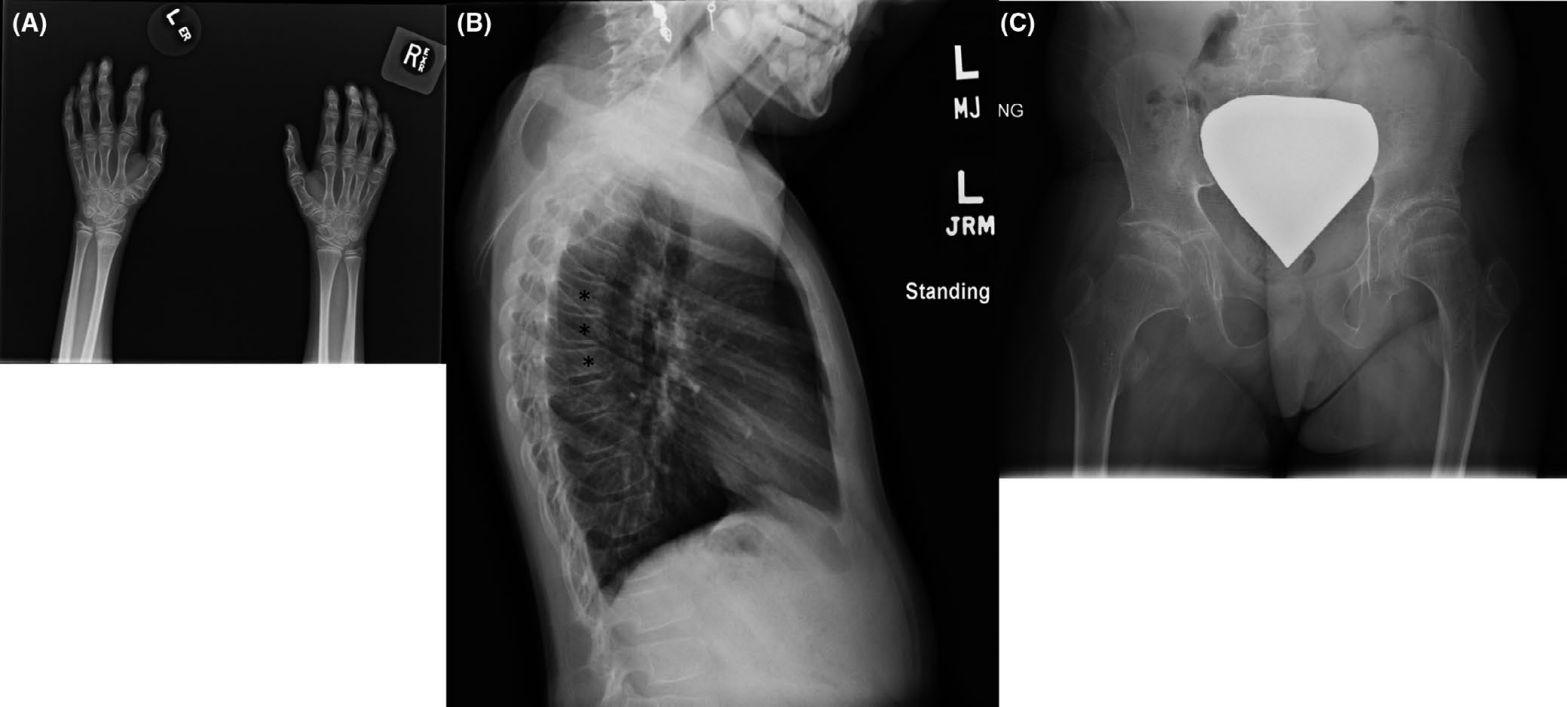
Radiograph of hands show the phalanges are shorter and broader, diffuse osteopenia, and narrowing of joint space in the wrists (A), spine shows platyspondyly indicated by asterisks (B) and hips show joint space narrowing, osteopenia, and erosion of the femoral heads (C)

**Figure 2:**
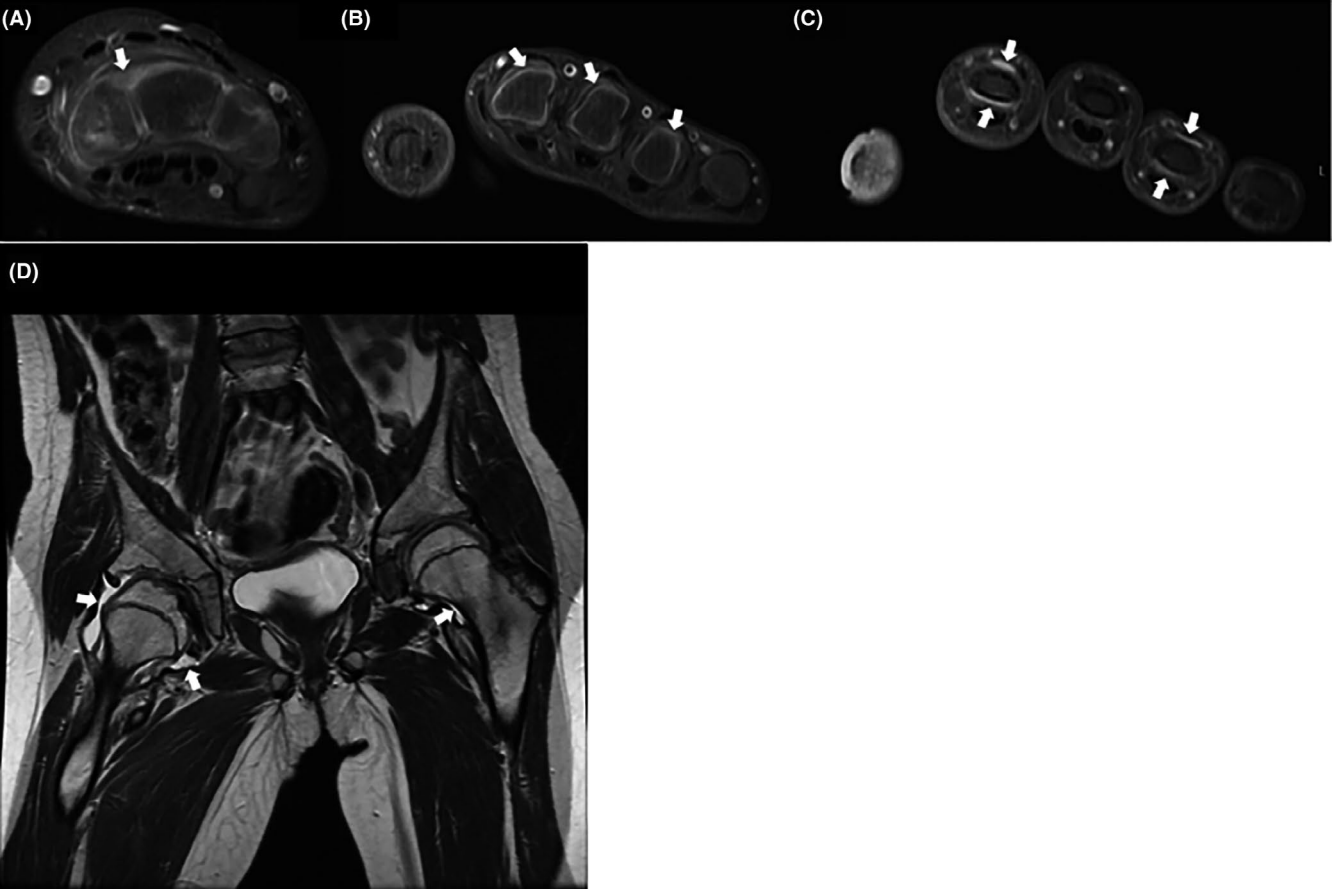
Axial contrast‐enhanced T1 fat‐saturated MR images show synovitis within the carpal joints (A), the MCP joints (B), and the PIP joints (C). (D) Coronal T2 MR image demonstrates large right hip and small left hip effusions, with irregularity of the right acetabulum and femoral head. MCP, metacarpophalangeal, PIP, proximal interphalangeal. Arrows indicate areas of synovitis

Patient 1, 9‐year‐old female, was referred to the pediatric rheumatology clinic at age 6 for evaluation of multiple joint swelling, bony hypertrophy, and joint contractures. The pregnancy and birth were unremarkable. Patient 1 was initially diagnosed with Juvenile Idiopathic Arthritis (JIA) but had atypical findings, including short stature. Radiograph of the hands revealed short phalanges with broad metaphysis, diffuse osteopenia, and narrowing of the joint space. Sagittal view spinal radiographs showed evidence of platyspondyly, and radiographs of the hips show osteopenia, joint space narrowing, and erosion of the femoral heads (Figure 1). Active inflammatory changes in her carpal, metacarpophalangeal (MCP), and the second through fifth proximal interphalangeal (PIP) joints of her left hand, which progressed over time to osseous erosions, were noted on MRI (Figure 2). Patient 1 showed active arthritis, with large right hip effusion, tiny effusion in the left hip, and what appeared to be chronic subarticular areas of sclerosis and minimal hyperemia both on the acetabular and femoral side of the joint. Patient 1 had no history of fractures. Blood test results revealed elevated erythrocyte sedimentation rate (ESR) of 45 mm/h, suggesting underlying inflammation. Total alkaline phosphatase of 200 IU/L and creatine phosphokinase (CPK) of 43 IU/L were within range. She was treated with numerous medications, beginning with traditional disease‐modifying antirheumatic drugs (DMARD), and then progressing to various biologic agents, without significant improvement. Patient 1 underwent radiographic and urine metabolic screening for mucopolysaccharidosis, with negative results.

**Figure 3:**
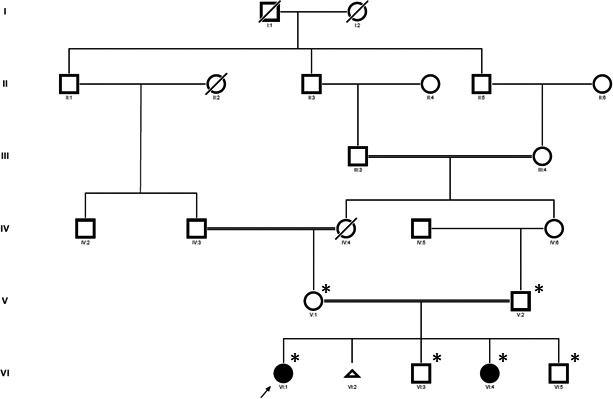
Family pedigree. The proband is indicated with an arrow. Asterisks denote family members that were included in the WGS study. WGS, whole‐genome sequencing

Patient 2 was a 5‐year‐old girl who presented at age 4 with mild joint limitations and bony hypertrophy that was localized to her cervical spine, as well as 4 focal PIP joints. Radiographs of both hands and her cervical spine were otherwise unremarkable (not shown). MRI of her cervical spine did not reveal signs of inflammation (not shown). Ultrasound of knees showed mild bilateral synovial thickening but no effusion or hyperemia. Blood test relieved ESR of 18 mm/h, total alkaline phosphatase of 222 IU/L, and creatine kinase of 91 IU/L. She was treated with nonsteroidal anti‐inflammatory drugs (NSAID) medications alone. Parents and brothers did not exhibit any symptoms. No other relevant family history of this disease was reported as shown in the pedigree (Figure 3).

Informed consent to participate in genetic studies was obtained from each participating family member as approved by the Institutional Review Board of The University of Texas Southwestern Medical Center at Dallas. Blood was collected by venipuncture, and DNA was purified by standard methods. DNA samples were processed at North Texas Genome Center at the University of Texas Arlington using the Illumina Nextera DNA Flex Library Prep Kit. Resulting libraries were sequenced with massively parallel sequencing by synthesis (SBS) chemistry on an Illumina NovaSeq 6000 sequencing system with 150 bp paired‐end reads. Reads were mapped and aligned, and variants were identified against the human GRCh37 reference genome build using the Edico Genome DRAGEN Germline Pipeline software v.3.0.5. The average coverage over the entire genome for the 6 samples sequenced was 55X. We used ANNOVAR to annotate allele frequency as found in the Genome Aggregation Database (gnomAD), nucleotide position within genes, and mutation type whether it was novel, deletion or insertion, nonsynonymous single nucleotide variant, stop‐gain, or stop‐loss, for each variant.^7^ Due to consanguinity in the family, we applied a recessive inheritance model to the resulting variants, selecting genotypes that were homozygous in both affected subjects, heterozygous in both parents, and never homozygous in controls. Candidate mutations were further restricted to those that had no more than a 1% minor allele frequency (MAF) in a general population (gnomAD) and altered the coding sequence (missense, nonsense, splice‐site, frameshift, and nonframeshift indel). ROHcaller implemented in BaseSpace^®^ App Whole Genome Sequencing v6.0 from illumine^®^ was used to calculate runs of homozygosity (ROH). Ideograms for ROH segments > 3 Mb were visualized in karyoploteR package.^8^

The novel homozygous *WISP3* (NM_198239c.930delinsAC, p.Thr312HisfsTer9) mutation identified by this analysis was confirmed by Sanger sequencing. Primers flanking *WISP3* exon 5 were designed using Primer3 (URL: http://primer3.ut.ee/); forward primer 5′TGCTGGAAATCACTACATAGCA3′; reverse primer 5′AAAGTAGATTTGCCACCATTTTA3′. PCR conditions are available upon request. PCR amplicon samples were further purified using ExoSAP‐IT PCR product cleanup reagent (Thermo Fisher, 78201.1.ML). Sanger sequencing was performed using the forward primer (Figure S1).

## 3 RESULTS

At 3.5 years of age, the proband in this family (Patient 1) initially presented in the neuromuscular clinic and was later referred to rheumatology when joint contractures were noted. The presence of swelling and limitation in multiple joints prompted an initial diagnosis of juvenile idiopathic arthritis (JIA). Her younger sister (Patient 2) later presented with similar symptoms and was also diagnosed with JIA. Patient 2 additionally reported multiple incidences of fractures of wrist, clavicle, and lower extremity secondary to falls. Given the family history of presumed JIA and consanguinity, the sisters were referred to genetics, where they were evaluated for dysostosis multiplex with a skeletal survey and urine mucopolysaccharide screen. The skeletal survey was found to be fairly unremarkable, with the exception of enlargement of interphalangeal joints and mild functional limitation. Urine mucopolysaccharides that were mildly out of range, but felt to be clinically unremarkable. Although genetic factors were suspected, it was recommended that available genetic testing at the time was unlikely to be informative.

The family was subsequently enrolled into a research protocol to perform whole‐genome sequencing. Sequencing family members identified recessively inherited, rare homozygous SNVs in the *ALKBH3*, *ARHGAP1*, *MYBPC3*, *TTC9C*, *PITPNM1*, *VWDE*, *CDC40*, *SMPDL3A*, and *WISP3* genes (Table S1). Of these, only one SNV encoded a predicted protein‐truncating mutation, in the *WISP3* gene, while the remaining mutations encoded missense changes. Mutations in the *WISP3* (Wnt‐1 Inducible Signaling Pathway Protein 3, MIM #603400) gene have been associated with progressive pseudorheumatoid dysplasia.^3^, ^9^, ^10^, ^11^, ^12^, ^13^ While the WISP3 Thr312HisfsTer9 mutation was a compelling candidate, given the consanguinity in the family it was possible that other possibly noncoding homozygous mutations could contribute to the disease. To test this, we analyzed the WGS data across the genome to search for regions for which the parents and unaffected sibling were heterozygous and the two siblings were homozygous. This analysis yielded four regions with ROH > 3 Mb, on chromosomes 3, 6, 7, and 11, that were shared in the affected but not the unaffected sibling (Figure S2). The chromosome 3 ROH was shared with the unaffected mother and therefore unlikely to contribute to disease. The *WISP3* candidate gene was contained within the 17.7 Mb ROH segment on chromosome 6. Thus, although we cannot fully interpret the contribution of homozygous noncoding mutations in the chromosome 7 or 11 ROH, or other smaller regions, we conclude that *WISP3* loss of function is most likely causal in this family.

## 4 DISCUSSION

PPD is a rare disorder with an incidence of 1/1 000 000 in the UK and predominately reported from the Middle East, Gulf States, and Turkey regions.^10^, ^14^ PPD is an autosomal recessive disorder that primarily affects articular cartilage.^11^ Protein‐altering mutations throughout the *WISP3* gene have been associated with PPD.^9^, ^11^ A recent review summarizes 66 PPD‐associated *WISP3* mutations from the literature.^9^ Of these, the majority of mutations predicted missense (~40%) or frameshift (~35%) changes to the amino acid sequence.^9^ Inflammation has not been noted in children diagnosed with PPD. However, one report does indicate an increase in inflammatory parameters in adult patients presumed to be due to cartilage degradation.^11^ Neither the depletion nor overexpression of *Wisp3* in mice confers a pathological phenotype, suggesting that, in contrast to humans, this protein is not essential for skeletal development or homeostasis. Overexpression of zWisp3 in zebrafish has been shown to inhibit bone morphogenetic protein (BMP) and Wnt signaling during development,^12^, ^13^ but whether these pathways contribute to the symptoms of PPD in humans is unclear.

## 5 CONCLUSION

PPD is a rare genetic disorder that mimics JIA. Imaging in the family revealed not only effusion but signs of active inflammation that are characteristic of JIA but not previously associated with PPD, to our knowledge. Thus, standard clinical evaluations originally confounded the diagnosis in this family. Our WGS approach illustrates the power of “clan genetics” married with genomics to reach an otherwise elusive diagnosis and inform treatment.^15^ In this instance, the family was counseled about their results, and unnecessary medications were discontinued.

## CONFLICT OF INTEREST

The authors declare no conflict of interest.

## AUTHOR CONTRIBUTIONS

CP: involved in the conception and design of the study, analyzed and interpreted the data, and wrote the manuscript. AK: analyzed and interpreted the WGS data, and wrote the manuscript. DW: analyzed and interpreted radiographic data. JR: involved in the conception and design of the study. KS: analyzed the WGS data. LN: involved in the conception and design of the study, analyzed, and interpreted the clinical data. CW: involved in the conception and design of the study, wrote the manuscript, and approved the final manuscript.

## ETHICAL APPROVAL

Written informed consent was obtained from all persons who participated in this study. This study was approved by the Institutional Review Board of The University of Texas Southwestern Medical Center at Dallas under study #: STU092011‐034.

## Supporting information

Fig S1

Fig S2

Table S1

## References

[1] Kim KH , Kim DS . Juvenile idiopathic arthritis: diagnosis and differential diagnosis. Korean J Pediatr. 2010;53(11):931‐935. 21218014 10.3345/kjp.2010.53.11.931 PMC3012272

[2] Al‐Mayouf SM . Noninflammatory disorders mimic juvenile idiopathic arthritis. Int J Pediatr Adolesc Med. 2018;5(1):1‐4. 30805524 10.1016/j.ijpam.2018.01.004 PMC6363254

[3] Ekbote AV , Danda D , Kumar S , Danda S , Madhuri V , Gibikote S . A descriptive analysis of 14 cases of progressive‐psuedorheumatoid‐arthropathy of childhood from south India: review of literature in comparison with juvenile idiopathic arthritis. Semin Arthritis Rheum. 2013;42(6):582‐589. 23270760 10.1016/j.semarthrit.2012.09.001

[4] Lifton RP . Individual genomes on the horizon. N Engl J Med. 2018;379(14):1353‐1362. 20220178 10.1056/NEJMe1001090

[5] Adams DR , Eng CM . Next‐generation sequencing to diagnose suspected genetic disorders. N Engl J Med. 2018;379(14):1353‐1362. 30281996 10.1056/NEJMra1711801

[6] French CE , Delon I , Dolling H , et al. Whole genome sequencing reveals that genetic conditions are frequent in intensively ill children. Intensive Care Med. 2019;45(5):627‐636. 30847515 10.1007/s00134-019-05552-x PMC6483967

[7] Wang K , Li M , Hakonarson H . ANNOVAR: functional annotation of genetic variants from high‐throughput sequencing data. Nucleic Acids Res. 2010;38(16):e164. 20601685 10.1093/nar/gkq603 PMC2938201

[8] Gel B , Serra E . karyoploteR: an R/Bioconductor package to plot customizable genomes displaying arbitrary data. Bioinformatics. 2017;33(19):3088‐3090. 28575171 10.1093/bioinformatics/btx346 PMC5870550

[9] Pode‐Shakked B , Vivante A , Barel O , et al. Progressive Pseudorheumatoid Dysplasia resolved by whole exome sequencing: a novel mutation in WISP3 and review of the literature. BMC Med Genet. 2019;20(1):53. 30922245 10.1186/s12881-019-0787-x PMC6439983

[10] Delague V , Chouery E , Corbani S , et al. Molecular study of WISP3 in nine families originating from the Middle‐East and presenting with progressive pseudorheumatoid dysplasia: identification of two novel mutations, and description of a founder effect. Am J Med Genet A. 2005;138A(2):118‐126. 16152649 10.1002/ajmg.a.30906

[11] Garcia Segarra N , Mittaz L , Campos‐Xavier AB , et al. The diagnostic challenge of progressive pseudorheumatoid dysplasia (PPRD): a review of clinical features, radiographic features, and WISP3 mutations in 63 affected individuals. Am J Med Genet C Semin Med Genet. 2012;160C(3):217‐229. 22791401 10.1002/ajmg.c.31333

[12] Nakamura Y , Weidinger G , Liang JO , et al. The CCN family member Wisp3, mutant in progressive pseudorheumatoid dysplasia, modulates BMP and Wnt signaling. J Clin Invest. 2007;117(10):3075‐3086. 17823661 10.1172/JCI32001 PMC1964511

[13] Kutz WE , Gong Y , Warman ML . WISP3, the gene responsible for the human skeletal disease progressive pseudorheumatoid dysplasia, is not essential for skeletal function in mice. Mol Cell Biol. 2005;25(1):414‐421. 15601861 10.1128/MCB.25.1.414-421.2005 PMC538768

[14] Teebi AS , Awadi SAA . Spondyloepiphyseal dysplasia tarda with progressive arthropathy: a rare disorder frequently diagnosed among Arabs. J Med Genet. 1986;23(2):189‐191. 10.1136/jmg.23.2.189-a PMC1049586 3712405

[15] Lupski JR , Belmont JW , Boerwinkle E , Gibbs RA . Clan genomics and the complex architecture of human disease. Cell. 2011;147(1):32‐43. 21962505 10.1016/j.cell.2011.09.008 PMC3656718

