# Supplementary figures and images for: Novel homozygous variant in WISP3 in a family with unrecognized progressive pseudorheumatoid dysplasia
Source: Clin Case Rep. 2020 May 3;8(8):1452–7. doi: 10.1002/ccr3.2884 (PMC7455413; doi:10.1002/ccr3.2884)

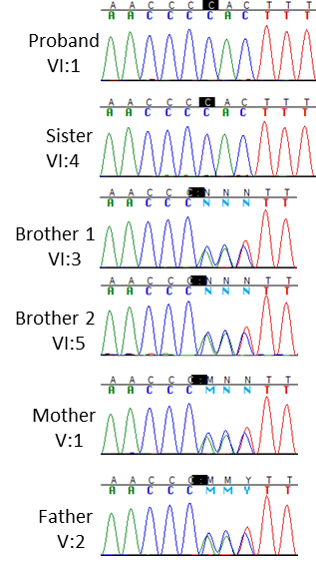

Supplement: Supplementary file 1 — Fig S1 [file CCR3-8-1452-s001.TIF]

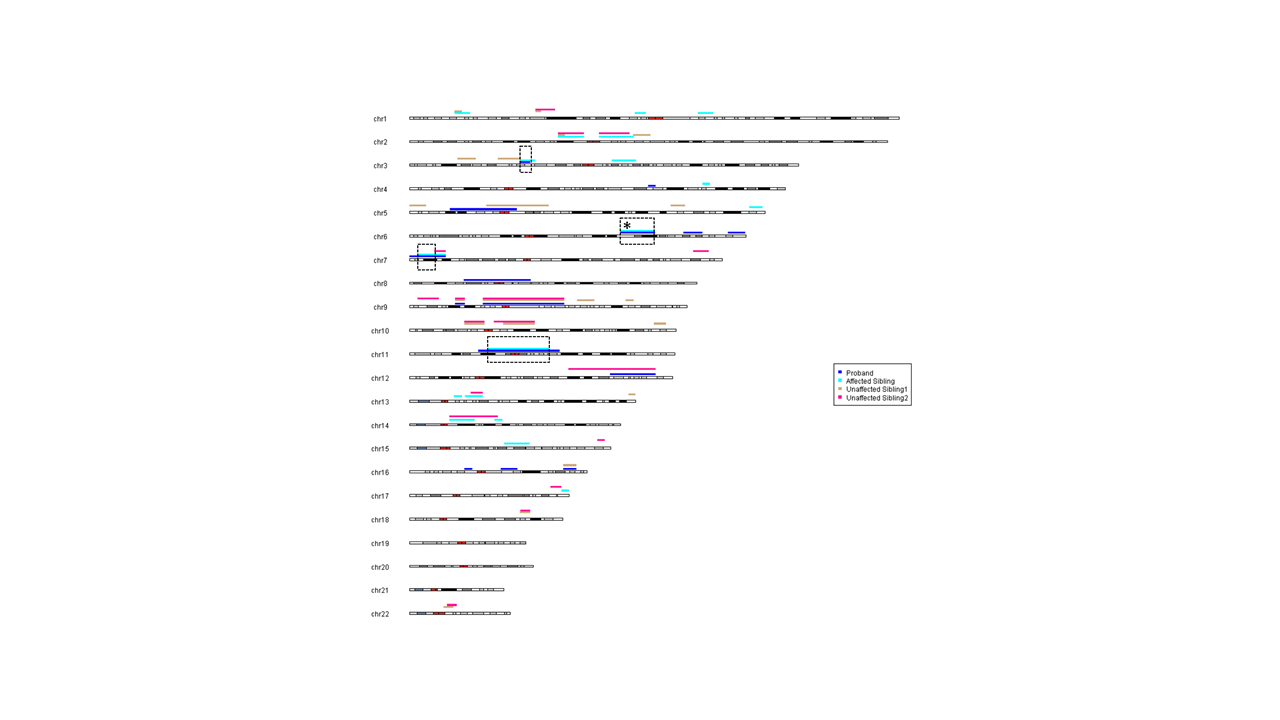

Supplement: Supplementary file 2 — Fig S2 [file CCR3-8-1452-s002.TIF]
